# Identifying environmental drivers of benthic diatom diversity: the case of Mediterranean mountain ponds

**DOI:** 10.7717/peerj.8825

**Published:** 2020-03-24

**Authors:** Saúl Blanco, Adriana Olenici, Fernando Ortega, Francisco Jiménez-Gómez, Francisco Guerrero

**Affiliations:** 1 Departamento de Biodiversidad y Gestión Ambiental, Facultad de Ciencias Biológicas y Ambientales, Universidad de León, Leon, Spain; 2 Babes-Bolyai University, Faculty of Environmental Science and Engineering, Cluj-Napoca, Romania; 3 Departamento de Biología Animal, Biología Vegetal y Ecología, Universidad de Jaén, Jaén, Spain; 4 Centro de Estudios Avanzados en Ciencias de la Tierra, Jaén, Spain

**Keywords:** Uniqueness, Isolation, Distance-decay, Elevation, Hydroperiod, Dispersal

## Abstract

This study aims at elucidating the environmental factors controlling benthic diatom diversity and uniqueness in Mediterranean mountain ponds. Samples of periphytic diatoms were collected in 45 ponds in Andalusia, south of Spain, and analysed by standard methods. Data analysis reveals that diatom diversity is mainly controlled by elevation and hydroperiod. Contrary to the usual findings in the literature, the highest scores on Shannon’s diversity index were found in high-elevation temporary ponds, but this effect is hidden by lake clustering in the analysed dataset. Significant distance-decay similarity (DDS) trends were detected in the analysis of floristic composition among the samples, stressing the importance of spatial factors that may override the effect of other abiotic factors. These findings highlight the role of isolation and dispersal limitation in the configuration of the biogeographical patterns of benthic diatoms.

## Introduction

Ponds are one of the most striking features in high elevation landscapes. Although these aquatic ecosystems represent a small area in mountainous regions, they are a key component in safeguarding aquatic biodiversity (Biggs et al., 2005; Martínez Sanz et al., 2012; Takaoka, 2015). Several studies have examined environmental factors influencing the biodiversity of high-elevation temperate lakes and ponds (Hinden et al., 2005; Catalan et al., 2006; Füreder et al., 2006; Robinson & Oertli, 2009; Martínez Sanz et al., 2012; Ilg & Oertli, 2014), but little attention has been paid to Mediterranean mountain systems (Escrivà, Armengol & Mezquita, 2010). In the Mediterranean region, these habitats are extremely sensitive to environmental stressors due to their long history of human settlement and impacts (which has led to significant alterations in ecological status), hence they play an important role for the assessment of the impact of environmental changes at a local level (Rosset, Lehmann & Oertli, 2010). Among the aquatic communities inhabiting these systems, diatoms are widely used to detect such alterations. In particular, periphytic diatoms constitute one of the most important components of algal assemblages in aquatic habitats in terms of diversity, biomass and ecosystem metabolism. However, the understanding of how diatom communities respond to environmental changes is still limited (Teittinen et al., 2016), and accordingly, the recent literature has a focus on identifying factors that control the diversity of diatoms in freshwater ecosystems (Marazzi et al., 2017).

Mountain ponds exhibit a high microbial phylogenetic singularity, constituting reservoirs of great evolutionary potential (Casamayor, 2017). These systems are now considered as biodiversity hotspots (Zaharescu et al., 2016). There are two scientific research lines in this regard; one line states that diatoms are ubiquitous and the composition of the community is determined by environmental conditions (Finlay & Fenchel, 1999; Finlay, 2002). Teittinen & Soininen (2015) found that dispersal limitations does not appear to influence diatom diversity at narrow scales, as species simply occur where their habitat requirements are met. The second line suggests that, on a larger scale, freshwater diatoms show biogeographic patterns that are related to dispersal processes, climate, and evolutionary history (Hillebr & Blenckner, 2002; Vanormelingen, Verleyen & Vyverman, 2008). According to Passy (2010), large-scale biodiversity patterns in freshwater protists are mostly resource-driven, compared to more complex organisms that are more strongly influenced by climate. These contrasting results demonstrate that different factors may affect species diversity at different spatial scales (Bolgovics et al., 2016); and hence, that climate operates at regional spatial scales while nutrients, pH and conductivity, among others, are factors that operate at the local scale (Teittinen & Soininen, 2015). In addition, it is commonly recognised that in regions with highly connected waterbodies, lentic diatom communities exhibit high diversity values (Vyverman et al., 2007; Soininen et al., 2009). This second line has been proposed as the best hypothesis with which to explain changes in diatom richness along the elevational gradient (Wang et al., 2011).

The exploration of elevational trends in diversity is essential for determining broad-scale distribution patterns (Soininen, 2012). In this sense, it becomes critical to understand the relative contribution of different environmental drivers impacting on taxa diversity, and how they connect to different scale gradients (Zaharescu et al., 2016).

Within this framework, Soininen & Teittinen (2019) have recently summarized the main open questions concerning the spatial ecology of diatoms. This paper answers specifically their question 1 dealing with the key factors explaining diversity patterns in diatoms. Particularly, the present study addresses the following questions: (i) Which is the main abiotic predictor of diatom diversity in mountain ponds? (ii) Are there dispersal limitations for diatom taxa in these ecosystems? (iii) Do rare taxa tend to occur in more isolated systems?

## Materials and Methods

**Figure 1:**
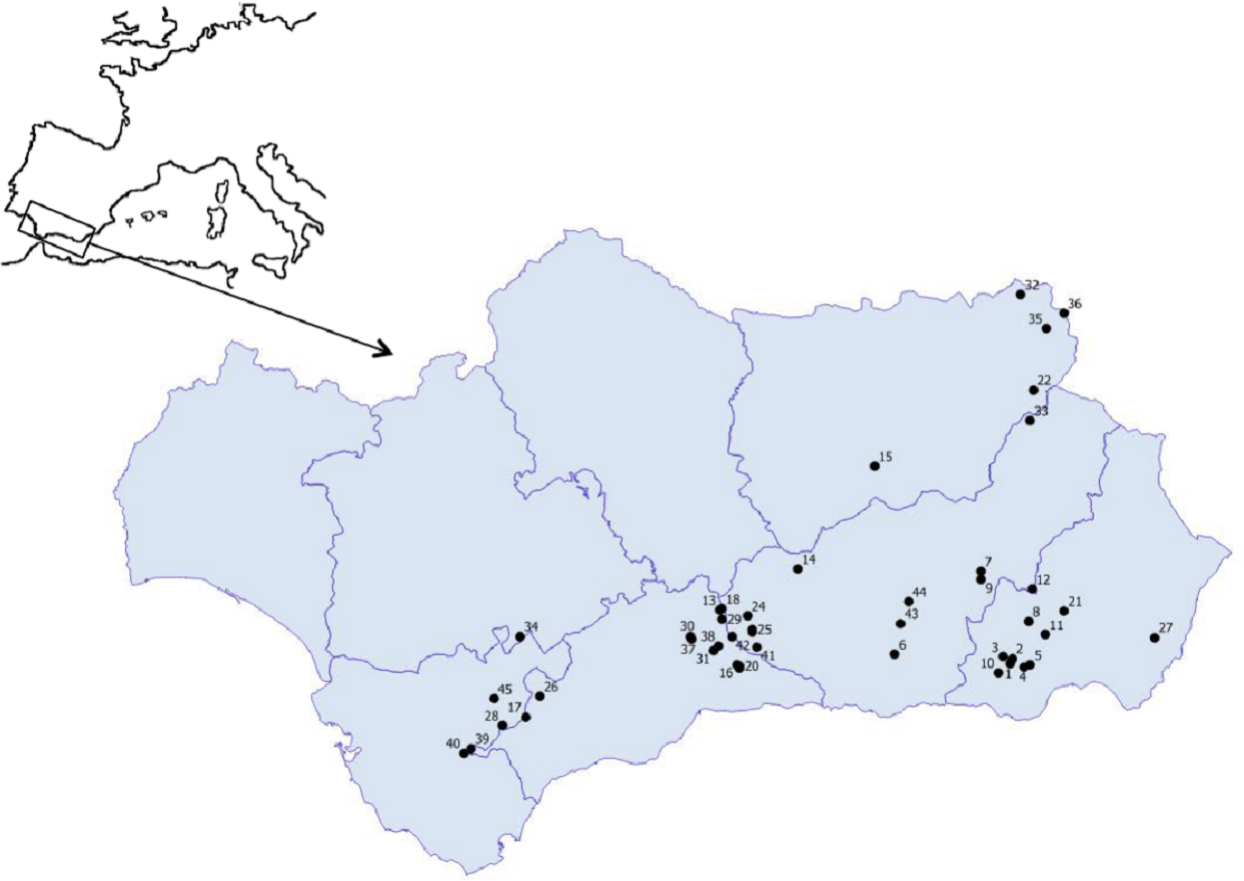
Geographic location of the studied ponds. **1.** Balsa Barjalí; **2.** Balsa Barroso; **3.** Balsa Blanca; **4.** Balsa Calabrial; **5.** Balsa Chanata; **6.** Balsa del Almiar; **7.** Balsa del Ciervo; **8.** Balsa del Entredicho; **9.** Balsa Pocico; **10.** Balsa del Sabinar; **11.** Balsa Salobre; **12.** Charca de Balax; **13.** Charca de Borbollobes Bajos; **14.** Charca de Juan Ramos; **15.** Charca de la Franciscuela; **16.** Charca de la Venta Marrullero; **17.** Charca de los Llanos de Líbar; **18.** Charca de Tolino; **19.** Charca del Cortijo de los Gabrieles; **20.** Charca del Cortijo del Perezón; **21.** Charca Filabres; **22.** Charco de la Tiná de las Cruces; **23.** Charco del Cornillejo; **24.** Charco del Negro; **25.** Charco del Nevazo Largo; **26.** Embalse de Bogas Bajas; **27.** Humedal de los Arejos; **28.** Laguna del Pazo; **29.** Laguna Chica de Archidona; **30.** Laguna de Caja; **31.** Laguna de Camarolos; **32.** Laguna de Castillo or Zarracotín; **33.** Laguna de Castril; **34.** Laguna de Coripe; **35.** Laguna de Orcera; **36.** Laguna de Siles or Bonache; **37.** Laguna de Viso; **38.** Laguna del Hondonero; **39.** Laguna del Moral; **40.** Laguna del Picacho del Algibe; **41.** Laguna del Rico; **42.** Laguna del Puerto de los Alazores; **43.** Lagunillo del Barranco de San Juan; **44.** Manantial de la Cuerda del Alguacil; **45.** Perezoso de la Loma de Albarracín Bajo.

During spring 2017 a biological survey was conducted (Junta de Andalucía approval no. SGYB/AF) in 45 mountain ponds, both temporary and permanent (median elevation: 1203 m a.s.l., range: 225–2520 m a.s.l.) located in Andalusia (south of Spain, Fig. 1). We consider temporary ponds those that permanently dried every year, whereas permanent ponds are those that do not dry for at least a decade (Chase, 2003). Our recent results (Rodríguez Alcalá et al., 2019) show that changes in diatom composition are mostly driven by conductivity and water depth; therefore, turbidity and conductivity were the unique environmental variables that were measured *in situ* since they are considered as excellent proxies of ecological status in continental lakes (Stenger-Kovács et al., 2018).

Periphytic diatoms were collected from each system and samples were processed and analyzed in the laboratory by following the method explained in Blanco & Becares (2006). Diatoms were cleaned and mounted on permanent microscope slides according to the European protocol EN14407. Due to the scarcity of diatoms in certain samples, a homogeneous count of *ca*. 100 individuals (diatom valves) were identified on each slide at the lowest possible taxonomic level following Hofmann, Werum & Lange-Bertalot (2011) and references therein. Counting 100 individuals has been found to be representative in terms of statistical reliability in ecological studies (Bate & Newall, 1998; Fatela & Taborda, 2002; Razumovskii, 2004). Uniqueness of diatom communities(*U*) of each pond *i* was calculated as:

\begin{eqnarray*}{U}_{i}=\sum _{j}{A}_{j} \left( 1-{O}_{j} \right) \end{eqnarray*}

where *A*_*j*_** and *O*_*j*_** are the abundance (relative) and the occurrence (rescaled to the [0–1] range), respectively, of the *j*th taxon. *U* values vary between 0 (all of the species of the sample are also found in all of the other ponds) and 100 (none of the taxa that were found occur in any other sample). Diatom diversity was calculated using Shannon–Wiener index:

\begin{eqnarray*}H=- \frac{\sum {n}_{i}}{n} ln \left( \frac{{n}_{i}}{n} \right) \end{eqnarray*}

where *n*_*i*_*/n* is the proportion of the *i*th taxon in the sample. Spatial clustering was tested with Nearest Neighbours analysis, whereas spatial autocorrelation was tested using Moran’s statistic. Finally, the degree of isolation (*I*) of each pond *i* was calculated with a Gaussian kernel density estimator (Hammer, Harper & Ryan, 2001):

\begin{eqnarray*}{I}_{i}=1- \left( \pi {r}^{-2}\sum _{i}{10}^{{ \left( -{d}_{i} \right) }^{2}2{r}^{-2}} \right) \end{eqnarray*}

where *d*_*i*_** is the Euclidean distance between pairs of locations, and *r* is the scale radius of the kernel, here set to 0.1.

To analyse the response of the diatom diversity and the uniqueness of diatoms with respect to abiotic factors, a Generalized Linear Model (GLM) ANCOVA model was calculated using hydroperiod and substrate type as categorical factors, and the other variables (limnological and geographical data) as continuous predictors. The resulting variables were selected based on a ‘best subsets’ algorithm (Neter et al., 1996). To compare the performance of the different models that were generated, we used Akaike’s Information Criterion (AIC).

Finally, distance matrices were computed using appropriate metrics (Sorensen’s index for floristic data, geographic distance for geographic data and Euclidean distance for elevational and limnological data) and distance decay of similarity (DDS) was evaluated by means of Partial Mantel tests (Legendre & Legendre, 1998) which consist on Pearson’s correlation tests between pairs of matrices based on random permutations.

Computations were performed by using Statistica (Statistica 13.0, StatSoft Inc., Tulsa, OK, USA) and PAST 3.19 (Hammer, Harper & Ryan, 2001) softwares. Raw data are available at DOI 10.6084/m9.figshare.11356262.

## Results

Most of the studied ponds (71% of the systems) were temporary, most of them (58%) presented limestone substrates, 22% presented Trias substrata and 20% presented siliceous substrata (Table 1). Pond waters were characterised by a wide range of mineralisation levels (0.03–10.40 mScm^−1^) and by moderate turbidity values (median: 11.32 NTU, range: 1.26–1000.00 NTU).

**Table 1:** Main environmental descriptors of he studied ponds.

Pond	Lat	Lon	Cond	Turb	Elevation	Hydroperiod	Substratum	S	Hα	Isolation	Uniqueness
Balsa Barjalí	36.9190	−2.7983	0.13	61.00	1713	Temporal	Lime	23	2.79	0.42	84.39
Balsa Barroso	36.9413	−2.7879	0.13	58.00	1542	Temporal	Lime	13	2.07	0.42	88.25
Balsa Blanca	36.9471	−2.8372	0.18	56.00	1551	Temporal	Lime	20	2.26	0.33	88.77
Balsa Calabrial	36.9052	−2.7226	0.28	67.00	1340	Permanent	Lime	11	1.44	0.69	73.06
Balsa Chanata	36.9142	−2.6916	0.29	131.00	1425	Temporal	Lime	16	1.97	0.69	75.80
Balsa del Almiar	36.9581	−3.4006	0.11	5.46	1775	Permanent	Siliceous	16	1.81	0.78	85.95
Balsa del Ciervo	37.3068	−2.9519	0.60	161.00	1630	Temporal	Lime	18	2.47	0.74	82.31
Balsa Entredicho	37.0967	−2.7011	0.23	420.00	1203	Temporal	Siliceous	18	2.28	0.57	83.98
Balsa Pocico	37.2708	−2.9475	1.56	2.60	1274	Permanent	Lime	15	1.81	0.74	82.09
Balsa Sabinar	36.8823	−2.8600	0.12	23.27	1830	Temporal	Lime	17	1.73	0.54	88.89
Balsa Salobre	37.0404	−2.6132	6.40	11.32	502	Permanent	Lime	10	1.30	0.75	93.72
Charca de Balax	37.2313	−2.6818	0.07	3.73	1892	Permanent	Siliceous	26	2.52	0.68	73.62
Charca de Borbollones Bajos	37.1369	−4.3184	1.40	3.99	723	Temporal	Trias	13	1.80	0.04	93.51
Charca de Juan Ramos	37.3143	−3.9124	0.29	3.51	1317	Permanent	Lime	24	2.70	0.91	76.98
Charca de la Franciscuela	37.7474	−3.5124	0.31	1.54	1339	Permanent	Lime	13	1.61	0.87	94.13
Charca de la Venta Marrullero	36.9103	−4.2257	0.60	21.96	457	Temporal	Trias	18	2.18	0.50	81.26
Charca de los Llanos de Líbar	36.6734	−5.3249	0.58	3.58	971	Permanent	Lime	7	0.69	0.75	97.19
Charca de Tolino	37.1419	−4.3080	0.14	124.00	727	Temporal	Trias	9	1.07	0.04	67.14
Charca del cortijo de los Gabrieles	36.8942	−4.2103	0.42	150.00	412	Temporal	Trias	8	0.75	0.50	90.31
Charca del cortijo del Perezón	36.9031	−4.2145	0.45	9.05	451	Temporal	Trias	13	1.66	0.50	71.33
Charca Filabres	37.1392	−2.5138	0.18	72.00	1049	Temporal	Siliceous	14	1.98	0.88	91.86
Charco de la Tiná de las Cruces	38.0681	−2.6733	0.42	41.27	1661	Permanent	Lime	9	1.67	0.82	87.84
Charco del Cornillejo	37.0479	−4.1468	0.27	51.00	1317	Temporal	Lime	31	2.72	0.16	79.50
Charco del Negro	37.1137	−4.1735	0.19	4.07	1476	Temporal	Lime	6	0.70	0.16	49.57
Charco del Nevazo Largo	37.0558	−4.1499	0.15	2.36	1347	Temporal	Lime	21	2.41	0.16	75.91
Embalse de Bogas Bajas	36.7612	−5.2503	0.15	5.35	750	Permanent	Siliceous	2	0.21	0.74	65.75
Humedal de los Arejos	37.0261	−2.0413	5.40	11.32	225	Permanent	Lime	17	1.40	0.92	93.77
Lagun del Pazo	36.6352	−5.4417	0.29	11.32	467	Temporal	Lime	19	2.32	0.82	84.59
Laguna Chica	37.0980	−4.3090	10.40	1.93	794	Permanent	Trias	15	1.89	0.04	91.20
Laguna de Caja	37.0215	−4.4696	0.86	137.00	728	Temporal	Trias	13	1.65	0.57	63.97
Laguna de Camarolos	36.9670	−4.3467	0.32	11.32	1336	Temporal	Lime	27	2.70	0.24	74.12
Laguna de Castillo	38.4670	−2.7360	0.29	11.32	783	Temporal	Trias	26	2.84	0.82	83.85
Laguna de Castril	37.9390	−2.6885	0.28	1000.00	1967	Temporal	Lime	13	1.58	0.90	88.23
Laguna de Coripe	37.0114	−5.3621	0.62	45.33	405	Temporal	Trias	8	0.66	0.86	49.10
Laguna de Orcera	38.3258	−2.6022	0.35	8.09	1263	Temporal	Lime	9	1.19	0.82	72.96
Laguna de Siles	38.3889	−2.5086	0.25	6.50	1288	Temporal	Lime	17	2.01	0.85	78.59
Laguna de Viso	37.0116	−4.4654	0.29	11.32	724	Temporal	Trias	2	0.16	0.57	81.81
Laguna del Hondonero	36.9873	−4.3215	0.33	3.90	1162	Temporal	Lime	10	1.36	0.06	67.35
Laguna del Moral	36.5354	−5.6023	0.18	1.26	664	Temporal	Siliceous	18	2.49	0.82	91.45
Laguna del Picacho del Algibe	36.5157	−5.6383	0.21	6.94	538	Temporal	Siliceous	11	1.62	0.82	93.04
Laguna del Rico	36.9831	−4.1207	0.34	8.17	895	Permanent	Lime	19	2.36	0.65	80.51
Laguna Puerto Alazores	37.0242	−4.2548	0.41	21.34	1039	Temporal	Lime	29	2.84	0.06	85.50
Lagunillo del Barranco de San Juan	37.0880	−3.3717	0.03	13.50	2520	Temporal	Siliceous	16	2.22	0.73	86.61
Manantial de la Cuerda del Alguacil	37.1786	−3.3312	0.03	1.63	2043	Temporal	Siliceous	15	2.31	0.85	89.62
Perezoso de la Loma de Albarracín Bajo	36.7452	−5.4916	0.29	11.32	728	Temporal	Lime	14	2.21	0.76	82.76

**Notes.**

Lat latitude Lon longitude Cond conductivity (mS cm^−1^) Turb turbidity (NTU)

A total of 238 diatom species (including subspecific taxa) were found, belonging to 64 different genera. Diatom assemblages were diverse (median Hα: 1.90, range: 0.16–2.84), and 25 species reached abundances>1% over the whole dataset. Although there was a statistically significant clustering in sampling locations (nearest neighbour analysis, *R* = 0.72, *p* < 0.001), no spatial autocorrelation was detected for diatom diversity along the distance gradient (Moran’s statistic, *p* > 0.05). Maximal richness was observed in Charco del Cornillejo, where 31 species were identified in a 100 valve count (Table 1). Dominant taxa included alkaliphilous adnate species such as *Achnanthidium pyrenaicum* or *Amphora pediculus*, and in general oligo-mesotrophilous taxa (*Achnanthidium minutissimum*, *Nitzschia perminuta*, *Fragilaria pararumpens*) were relatively abundant and widespread in the analyzed systems. Based on the low conductivity values that were observed, most of the ponds studied could be characterized as fresh to subsaline ecosystems with respect to the diatom communities they contain.

Average occurrence was 6.4%, with 47% of taxa only appearing in a single pond. On the contrary, the ubiquitous *Achnanthidium minutissimum* was present in 56% of the samples. Three unknown diatom species were found in these ponds, recently described by us as new taxa, e.g., Blanco et al. (2019). Maximal uniqueness was observed in Charca de los Llanos de Líbar, where 97% of the individuals that were identified belong to taxa not found elsewhere, with the occurrence of *A. jackii* having an abundance of 84%.

### GLM models

**Figure 2:**
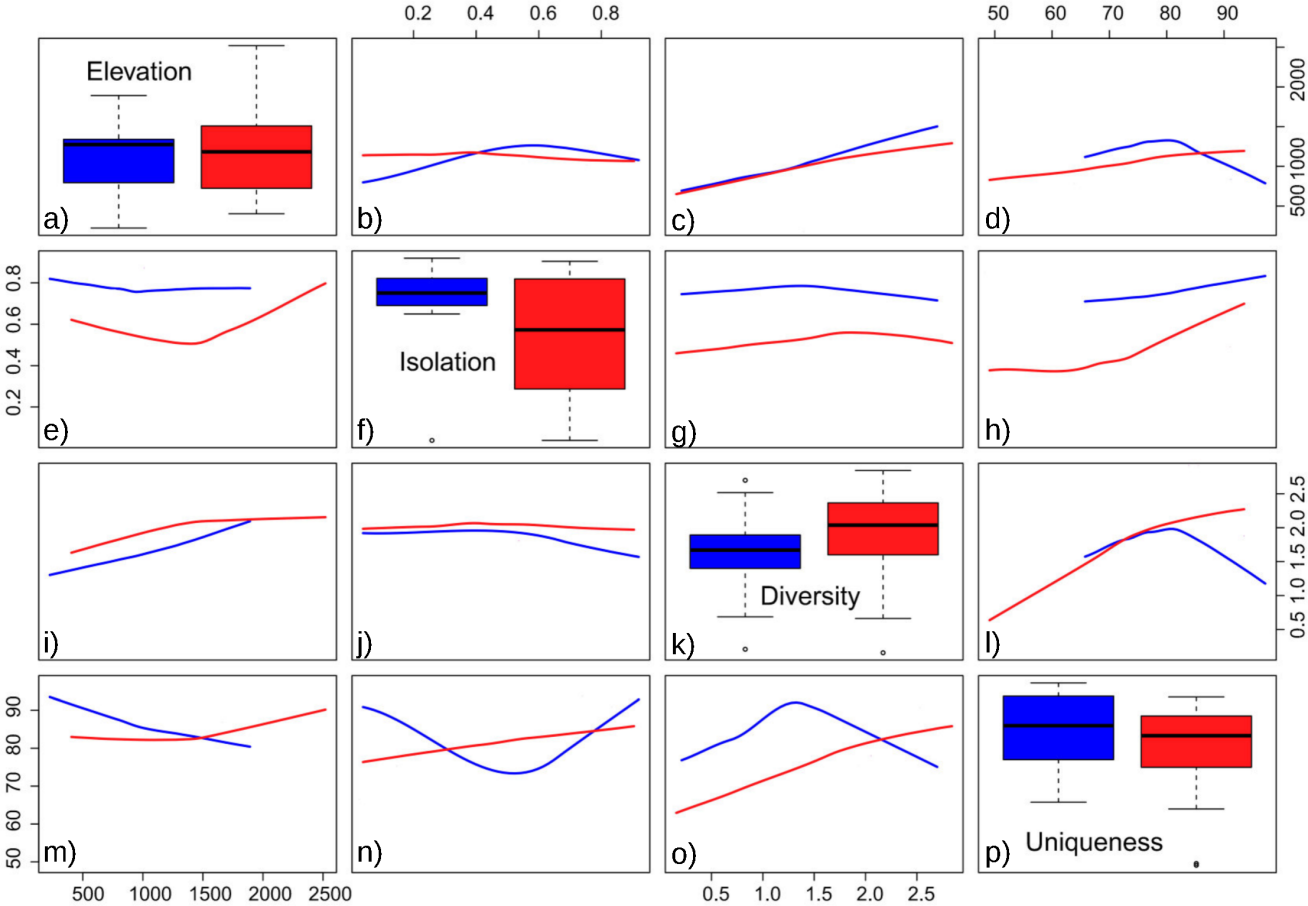
Pairwise relationships between elevation, isolation, diversity and uniqueness in the studied ponds. (A) Boxplot of elevation values. (B) Relationship between elevation and isolation. (C) Relationship between elevation and diversity. (D) Relationship between elevation and uniqueness. (E) Relationship between isolation and elevation. (F) Boxplot of isolation. (G) Relationship between isolation and diversity. (H) Relationship between isolation and uniqueness. (I) Relationship between diversity and elevation. (J) Relationship between diversity and isolation. (K) Boxplot of diversity. (L) Relationship between diversity and uniqueness. (M) Relationship between uniqueness and elevation. (N) Relationship between uniqueness and isolation. (O) Relationship between uniqueness and diversity. (P) Boxplot of uniqueness. Data (omitted) fitted to LOESS smoothers. Blue: permanent ponds. Red: temporary ponds.

**Figure 3:**
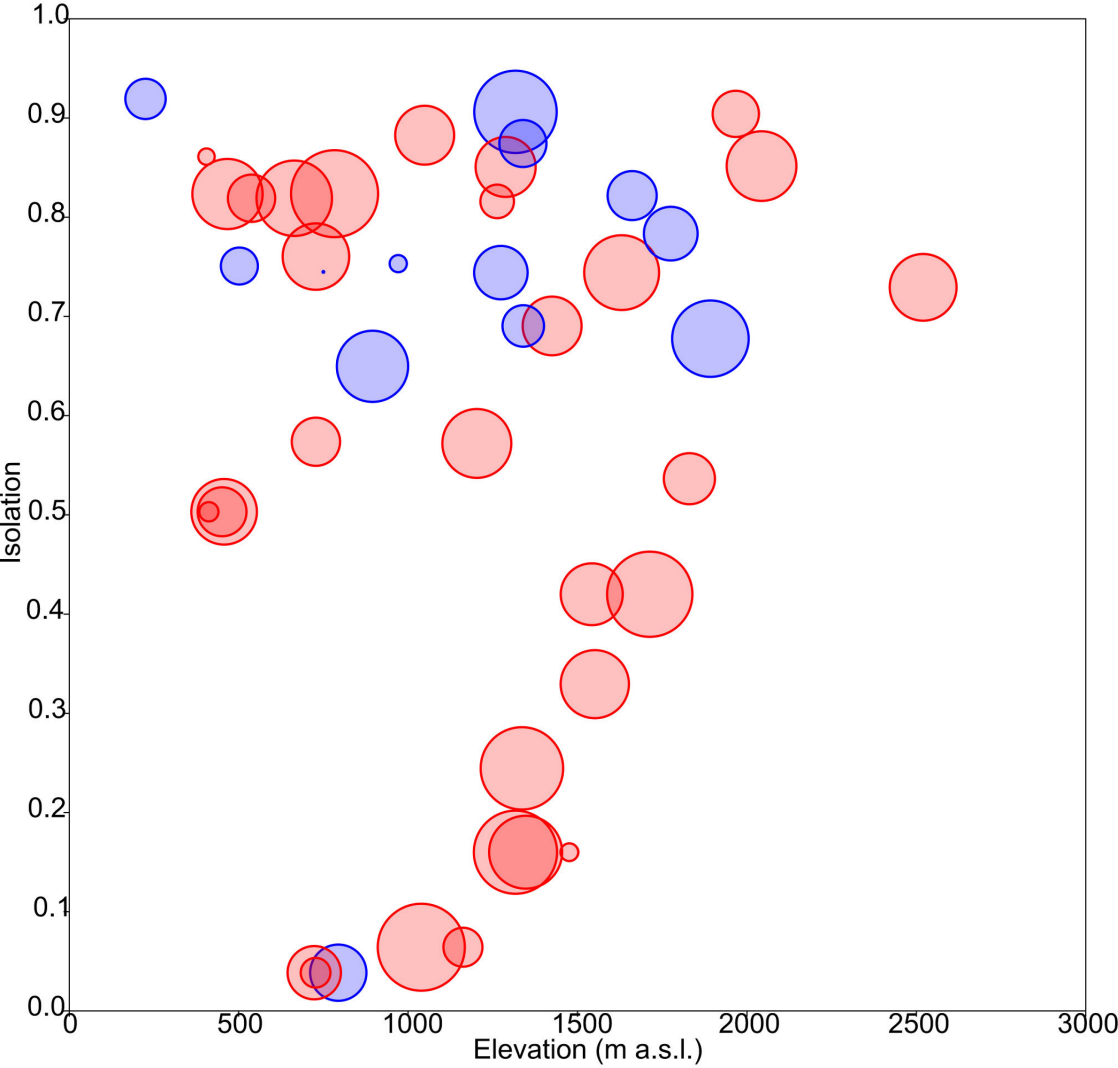
Response of diatom diversity to elevation and isolation. Circle diameter is proportional to Shannon’s *H*_*α*_ index. Blue: permanent ponds. Red: temporary ponds.

The obtained results showed the lowest AIC values for GLM models with a Gaussian error distribution and an identity link function. Three variables were selected as the best predictors for diatom diversity: elevation (*p* = 0.02), uniqueness (*p* = 0.03), and hydroperiod (*p* = 0.09), with no significant interactions. Figure 2 describes the relationships between these variables: diversity increases monotonically with elevation, and with uniqueness but only in temporary systems, where diversity values were higher on average. Isolation had no apparent relationship with diversity (Fig. 3).

**Figure 4:**
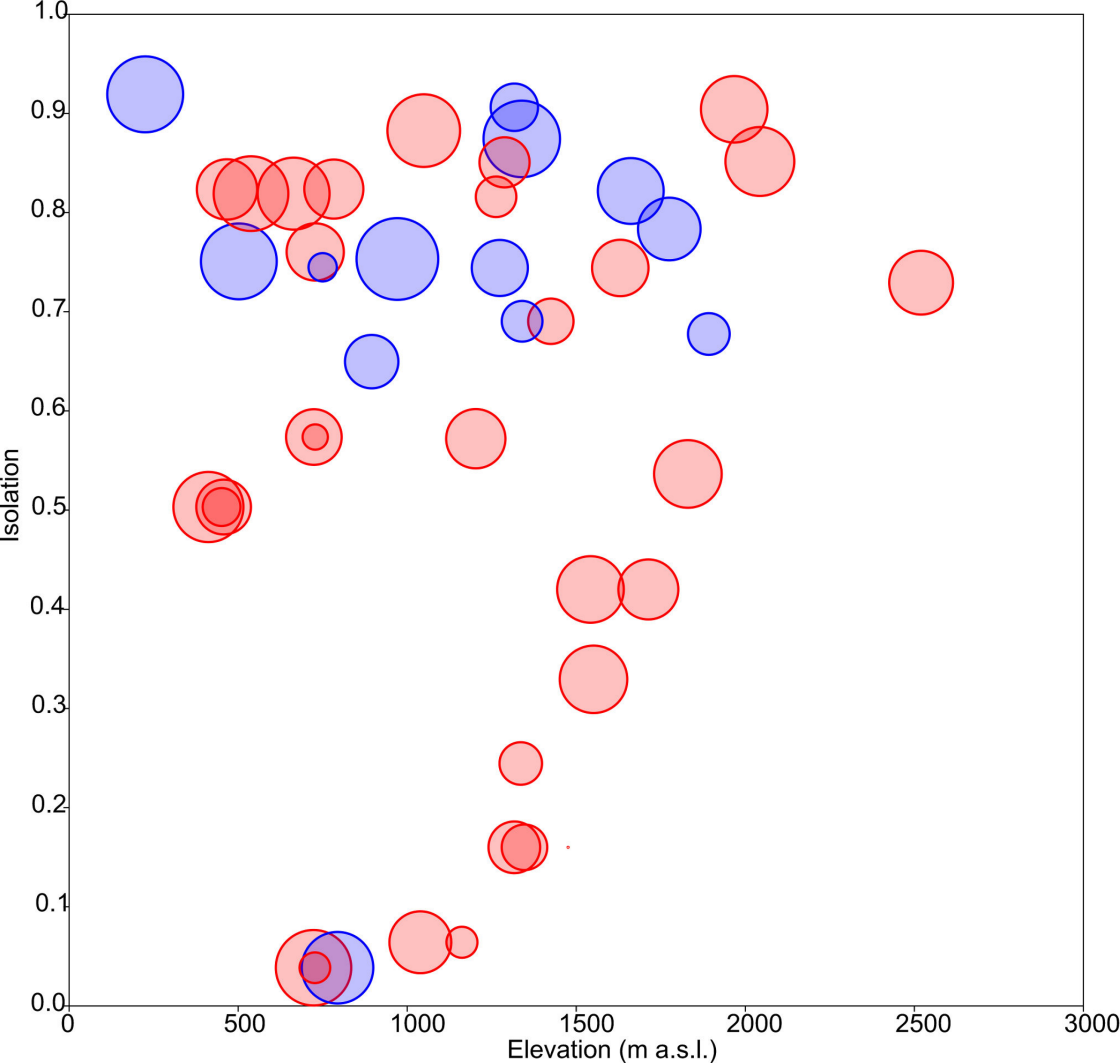
Response of floristic uniqueness to elevation and isolation. Circle diameter is proportional to uniqueness. Blue: permanent ponds. Red: temporary ponds.

Floristic uniqueness depended significantly on water conductivity (*p* = 0.01), isolation (*p* = 0.03) and diversity (*p* = 0.01). An increase in floristic uniqueness with increasing isolation (Fig. 4) and conductivity (data available at DOI 10.6084/m9.figshare.11356262) was only evident for temporal ponds. In general, variable relationships are monotonic in temporal systems, whereas unimodal patterns tend to occur in permanent systems (Fig. 2).

### Distance matrices

**Figure 5:**
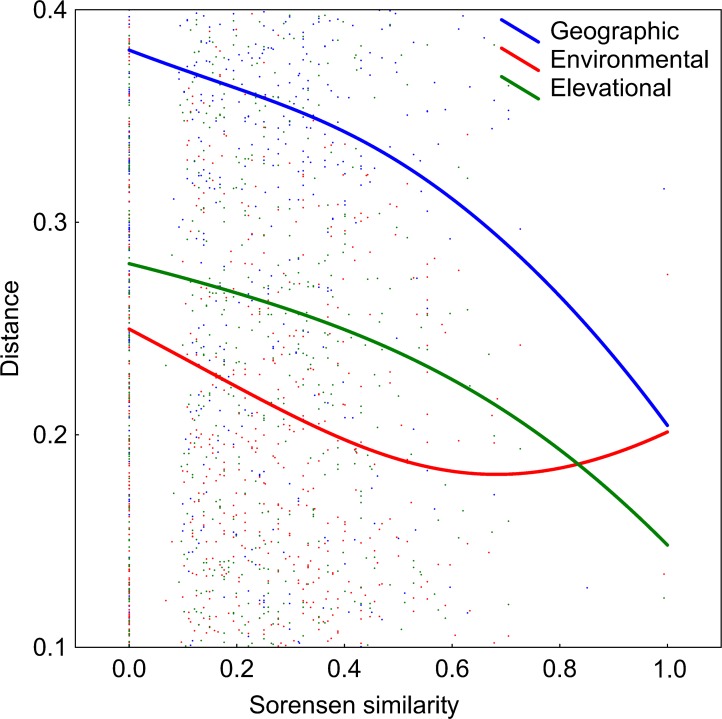
Relationships between elevational, environmental and geographic distances and Sorensen similarity in the analyzed systems. Data fitted to LOESS smoothers.

DDS trends are shown in Fig. 5. The number of co-occurring taxa (measured as Sorensen similarity) decreased with geographic distance. The community composition of ponds located at different altitudes was also dissimilar. Likewise, different environmental conditions also led to different diatom assemblages although, contrary to the other two previously mentioned relationships, the slope of this trend decreases along the environmental distance gradient. However, it can be seen in Table 2 that the observed decay in community similarity with increasing elevation is mostly due to the positive correlation between elevation and geographic distance in the analysed dataset (R^2^ = 0.2, p<0.01). Geographical distance also confounds the relationship between floristic and environmental distances, although DDS in Sorensen’s index remains statistically significant even when the environmental and elevational distances are taken into account.

### Uniqueness vs. isolation

**Figure 6:**
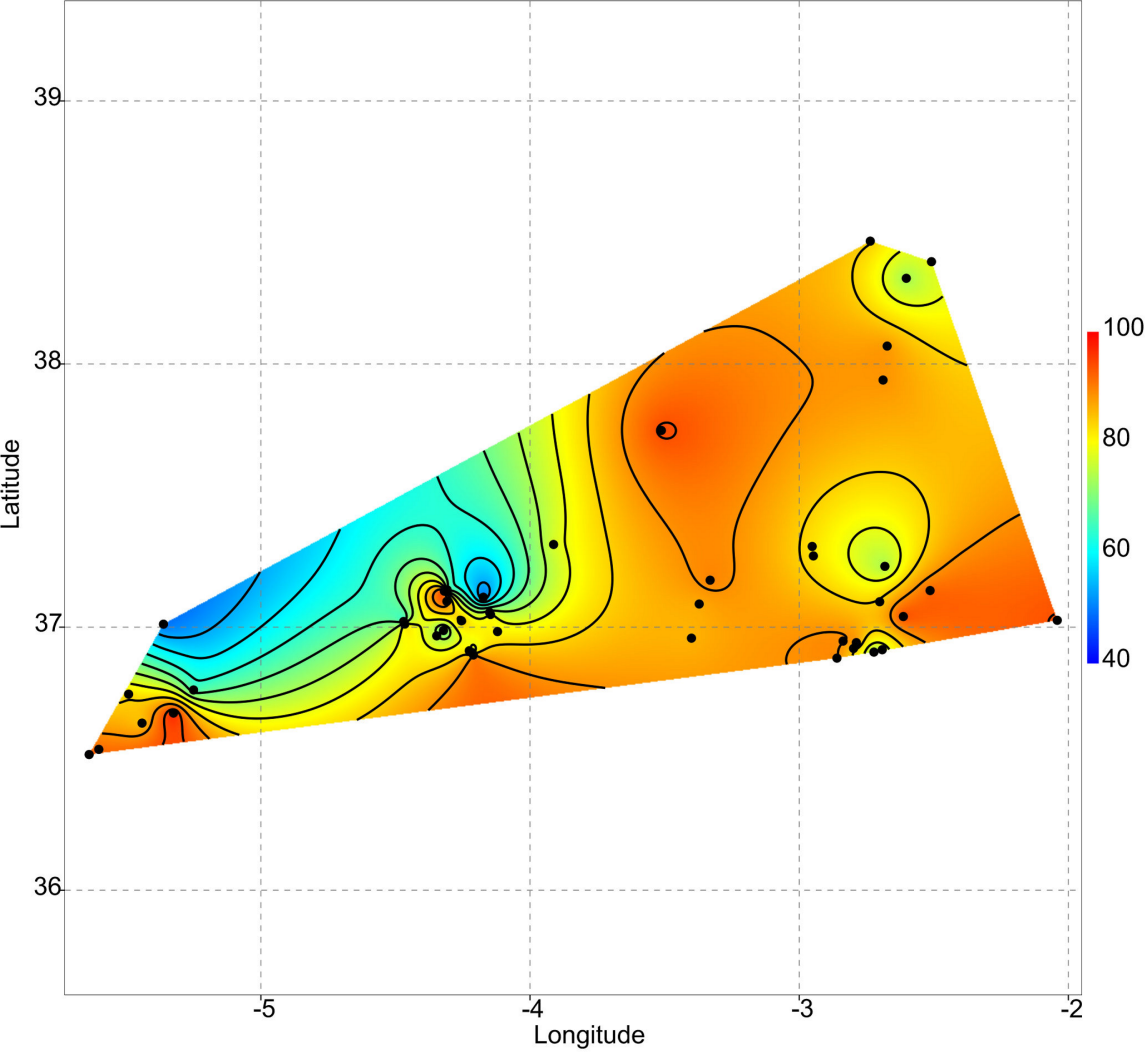
Geographic variation of floristic uniqueness in the sampling locations.

As aforementioned, rare taxa tend to occur in ponds that are more isolated. This can be evidenced by plotting the geographic distribution of uniqueness scores in the studied systems (Fig. 6). As a general rule, ponds with large average distances to the surrounding lakes contain low-occurrence taxa.

## Discussion

### Diatom diversity predictors in Andalusian mountain ponds

The results obtained in this study show that the most diverse diatom communities appear in high elevation ponds. The relationship between diatom diversity metrics and elevation has been previously analysed in the literature, but only a few publications report a clear and significant trend (Teittinen et al., 2016); only Wang et al. (2011) observed a monotonic decrease of diatom diversity with elevation in stony streams. The lack of general trends in this pattern suggests that diatom diversity could be more affected by local environmental factors rather than by climatic variables that are associated with elevation (Hillebrand & Blenckner, 2002). The presence of a dominant, constraining environmental variable in the dataset may also obscure this relationship (Teittinen et al., 2016). However, in our case, neither conductivity nor turbidity were observed to be significantly affecting diatom diversity. This contrasts with the literature consensus (Soininen & Heino, 2007; Teittinen & Soininen, 2015; Jyrkänkallio-Mikkola, Heino & Soininen, 2016) reporting on how different environmental variables affect this metric. At a regional scale, the effect of other variables on diatom diversity, such as pH or nutrients, has been found to be very weak or nonlinear (Wang et al., 2017). This is probably because historical factors explain significantly more regarding the observed diversity patterns than do the contemporary environmental conditions (Vyverman et al., 2007; Vilmi, Karjalainen & Heino, 2017). Therefore, although in high elevation areas the prediction is related to an increase of diatom richness as a consequence of the global change (Rosset, Lehmann & Oertli, 2010), human activities tend to yield water quality impairment that could be masking this pattern.

**Table 2:** Partial Mantel tests results comparing the floristic distance matrix (Sorensen index) with the other distance matrices calculated.

Compared matrix	When controlling for similarities given in:	R	*p* value
Elevational	Environmental	0.03	<0.01
Elevational	Geographical	0.07	0.80
Environmental	Elevational	0.08	<0.01
Environmental	Geographical	0.10	0.31
Geographical	Elevational	0.07	<0.01
Geographical	Environmental	0.07	<0.05

### Diatom distance patterns

The obtained results indicate that diatom diversity is higher in temporary ponds, a trend that is observed in Mediterranean wetlands in other biological groups (Gilbert et al., 2014). These systems usually support different assemblages, hosting species that are endemic or rare at regional scales (Pérez-Bilbao, Benetti & Garrido, 2015). For instance, several new diatom species have been found to inhabit these ponds (Blanco et al., 2019). However, the majority of cases in the literature indicate that permanent ponds harbor more diverse communities than temporary ponds (Bouchard, Gajewski & Hamilton, 2004; Anton-Pardo, Armengol & Ortells, 2015; Bolgovics et al., 2016). In the present study, it can be seen that floristic uniqueness does not change with hydroperiod, but rather it changes with respect to isolation degree (Fig. 3), as the temporary lakes tend to appear more spatially grouped. This may have contributed to increases in diatom richness through dispersal, which is consistent with DDS trends that are observed in floristic similarity (i.e., nearby ponds have similar species composition). It has been shown that species assemblages are likely to be richer in areas that facilitate propagule dispersal and colonisation (Zaharescu et al., 2016). This idea is clearly related to the metacommunity and metaecosystems theory (Loreau, MOuquet & Holt, 2003; Leibold et al., 2004). In fact, our data reveal a significant relationship between geographic and floristic distances that cannot be attributed to elevational or environmental gradients (Table 2). The observed DDS (Fig. 5) suggests that (i) there are biotic and/or abiotic limitations that constrain organisms’ dispersal, and (ii) communities are controlled by niche-based processes. The same relationship has been suggested by Szabó et al. (2018) for pond diatoms, in which environmental factors are of minor importance compared with spatial factors (but see Teittinen & Soininen (2015). The calculated Mantel tests indicate that the observed decline in community similitude along the elevational and environmental gradients cannot be disentangled from the effect of geographic distance, suggesting that environmental filtering did not account for much of the among-site differences in community composition. This may be due to the relatively short environmental ranges in the dataset or to the overriding of spatially structured parameters (Teittinen et al., 2016).

### Floristic uniqueness

In the studied systems, more isolated ponds tend to be more unique in their taxonomic composition. Thus, isolation becomes an important variable affecting the ecological uniqueness of ponds (Vilmi, Karjalainen & Heino, 2017). Moreover, the positive relationship between diversity and uniqueness that is observed in temporary ponds (Fig. 2) supports the idea by Soininen & Heino’s (2007) that sites with high-diversity assemblages are likely to be occupied by specialists with a narrow niche breadth, whereas low diversity assemblages are dominated by generalists. On the contrary, Szabó et al. (2018) report that the ecologically most unique sites among Carpathian ponds hosted relatively low species richness. Such different results may be related to the degree of anthropogenic disturbance. In our study area, the finding of low-occurrence taxa in isolated ponds could indicate that broad-scale land use and hydrological alteration of the environment has not homogenised these assemblages (Winegardner et al., 2017).

## Conclusions

Although they are often excluded in conservation studies, microorganisms (e.g., periphytic diatoms) arise as essential components of biodiversity in mountain regions (Casamayor, 2017). We found that diatom diversity in mountain ponds essentially responds to geographic factors that mask any plausible contribution of environmental or elevational gradients. The link that is observed between isolation, uniqueness and diversity indicates that isolated ponds are clear targets if floristic singularities are a conservation goal (Vilmi, Karjalainen & Heino, 2017). In conclusion, periphytic diatom communities in the studied mountain ponds are likely to be driven by regional factors, and therefore are mostly shaped through dispersal limitation. Further studies with a focus on exploring the drivers of diatom diversity and uniqueness at smaller temporal and spatial scales are required.

## Additional Information and Declarations

### Field Study Permissions

The following information was supplied relating to field study approvals (i.e., approving body and any reference numbers): The authors would like to thank Junta de Andalucía for the permits that were granted for the sampling of mountain ponds in Andalusia (SGYB/AF).

### Data Availability

The following information was supplied regarding data availability: Raw data are available at Figshare: Blanco, Saul (2019): Raw data for “Identifying environmental drivers of benthic diatom diversity: the case of Mediterranean mountain ponds”. figshare. Dataset. DOI 10.6084/m9.figshare.11356262.v1.
